# Biochemical, Mineral, and Morphological Properties of Indian Tamarind (*Tamarindus indica* L.)

**DOI:** 10.1002/fsn3.71754

**Published:** 2026-04-12

**Authors:** Daya Shankar Mishra, Vikas Yadav, Lalu Prasad Yadav, Jagadish Rane, Varre Venkata Apparao, Kanupriya Chaturvedi, Prakashbhai Ravat, Prashant Kaushik, Prakash Kumar, Yazgan Tunç, Ali Khadivi

**Affiliations:** ^1^ Division of Crop Improvement ICAR‐CIAH RS Central Horticultural Experiment Station Godhra Gujarat India; ^2^ ICAR‐Central Institute for Arid Horticulture Bikaner Rajasthan India; ^3^ Division of Fruit Crops ICAR‐Indian Institute of Horticultural Research Bengaluru Karnataka India; ^4^ Department of Vegetable Science Chaudhary Charan Singh Haryana Agricultural University Hisar Haryana India; ^5^ ICAR‐Indian Agricultural Statistics Research Institute (IASRI) New Delhi India; ^6^ Republic of Türkiye, Ministry of Agriculture and Forestry, General Directorate of Agricultural Research and Policies Hatay Olive Research Institute Directorate Hassa Hatay Türkiye; ^7^ Department of Horticultural Sciences, Faculty of Agriculture and Natural Resources Arak University Arak Iran

**Keywords:** cluster analysis, correlation, PCA, tamarind, yield

## Abstract

Tamarind ( *Tamarindus indica* L.) is an economically important perennial fruit tree valued for its pulp yield, nutritional composition, and processing suitability. This study evaluated 10 grafted tamarind cultivars grown under semi‐arid conditions in Gujarat, India, over four seasons (2020–2024) to assess morphological, yield, biochemical, and mineral traits and to identify superior genotypes using multivariate approaches. Significant varietal differences (*p* < 0.001) were observed for most traits. Goma Prateek exhibited superior pod length (15.0 cm), pulp weight (9.21 g), vitamin C (24.1 mg 100 g^−1^), total phenols (67.9 mg 100 g^−1^), total flavonoids (35.5 mg 100 g^−1^), antioxidant activity (89.4%), calcium (0.75%), and iron (0.24%). Pratisthan showed the highest pulp percentage (49.8%) and real pulp value (5.13). Genotypic correlations revealed strong associations among pulp weight, real pulp value, pod weight, and mineral traits. Principal component analysis explained 98.90% of total variability in the first eight components, with PC1 (27.40%) associated mainly with yield traits and PC2 (20.60%) with pulp quality attributes. Cluster analysis grouped cultivars into three distinct clusters, highlighting substantial divergence; Sweet Type and Goma Prateek showed the greatest genetic distance. Overall, Goma Prateek, Ajanta, and Pratisthan emerged as promising cultivars combining yield and nutritional quality. The integrated evaluation of morphological, biochemical, and mineral traits provides a robust framework for tamarind breeding and cultivar selection under semi‐arid conditions.

## Graphical abstract

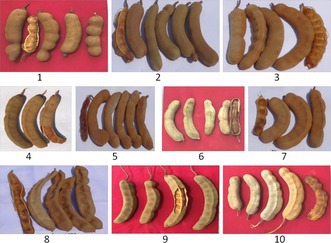

## 1 Introduction

Tamarind ( *Tamarindus indica* L.; 2n = 2× = 24), belonging to the Fabaceae family, is an economically significant fruit tree. It is native to Africa but widely cultivated across subtropical and tropical areas, especially in South Asia, Southeast Asia, and Latin America. Tamarind is a large, evergreen, and long‐lived tree and grows up to 30 m tall with pinnately compound leaves, yellow flowers streaked with red, and pod‐like fruits containing tangy pulp and seeds. Owing to its deep root system and adaptability, tamarind thrives under diverse soil and climatic conditions and is particularly suited to hot and semi‐arid regions (Singh et al. 2021, 2025).

The tamarind pod includes 25%–40% seeds, 11%–30% shell, and 30%–50% pulp. The pulp is a key ingredient in cuisines worldwide, imparting a sweet–sour flavor to dishes like sambhar, Pad Thai, and Tom Yum. It is also used in the production of beverages, confectioneries, and even alcoholic drinks, particularly in South America (Nagar et al. 2022; Akter et al. 2023; Mayavel et al. 2025). The pulp is processed into globally traded products such as concentrates, powders, sauces, and beverages, contributing significantly to international food markets (Nagar et al. 2022; Mayavel et al. 2025). Tamarind seeds are roasted and consumed as snacks, whereas tamarind kernel powder and gum are widely used as natural food thickeners and stabilizers in beverages, confectioneries, and pharmaceutical formulations (Nagar et al. 2022; Akter et al. 2023). Biochemically, tamarind pulp is rich in organic acids (notably tartaric acid), reducing sugars, phenolic compounds, flavonoids, and vitamin C, along with essential minerals such as potassium, calcium, magnesium, and iron (Kidaha et al. 2019; Komakech et al. 2019; Akter et al. 2023; Reddy et al. 2024). These constituents confer antioxidant, anti‐inflammatory, antimicrobial, and cardioprotective properties. The seeds, bark, and leaves have also been reported to possess significant antimicrobial and wound‐healing properties, as substantiated by experimental studies (Okello et al. 2017; Mishra 2018; Nagar et al. 2022). However, most previous studies have focused on compositional analysis of limited genotypes or specific regions, often evaluating either biochemical parameters or mineral composition independently, with minimal integration of these traits across diverse germplasm sets.

Despite its economic importance, systematic studies on genetic and phenotypic variability in tamarind remain limited. Predominant seed propagation and natural outcrossing have contributed to extensive variability (Kumar et al. 2023; Mayavel et al. 2025), which is reflected in fruit morphology, pulp yield, acidity, and compositional traits across regions (Van den Bilcke et al. 2014; Okello et al. 2017; Kidaha et al. 2019; Pooja et al. 2022). Characterization efforts using morpho‐agronomic descriptors (Pooja et al. 2022; Reddy et al. 2023; Kumar et al. 2023; Mayavel et al. 2025; Singh et al. 2025) and molecular markers (Kumar et al. 2015; Sarmiento et al. 2017; Kanupriya et al. 2024) have revealed substantial diversity; however, the comprehensive integration of agronomic, biochemical, and mineral traits for identifying nutritionally superior genotypes remains insufficiently explored.

Multivariate tools such as principal component analysis (PCA) and clustering are effective for classifying genotypes based on complex trait interactions and for identifying promising candidates for breeding and conservation (Álvarez et al. 2019; Pooja et al. 2022; Kanupriya et al. 2025). Understanding trait correlations and genetic distances is crucial to develop improved cultivars that support food security and sustainable agriculture. Nevertheless, few studies have applied these approaches to simultaneously evaluate fruit yield components alongside detailed biochemical and mineral profiling. Therefore, the present study addresses this gap by conducting an integrated evaluation of diverse Indian tamarind cultivars for agronomic, biochemical, and mineral traits, followed by multivariate analysis to elucidate trait interrelationships and identify superior genotypes with enhanced nutritional and commercial potential. This comprehensive approach provides a more holistic framework for tamarind improvement, germplasm utilization, and value‐based cultivar selection.

## 2 Materials and Methods

### 2.1 Study Site and Experimental Material

**FIGURE 1:**
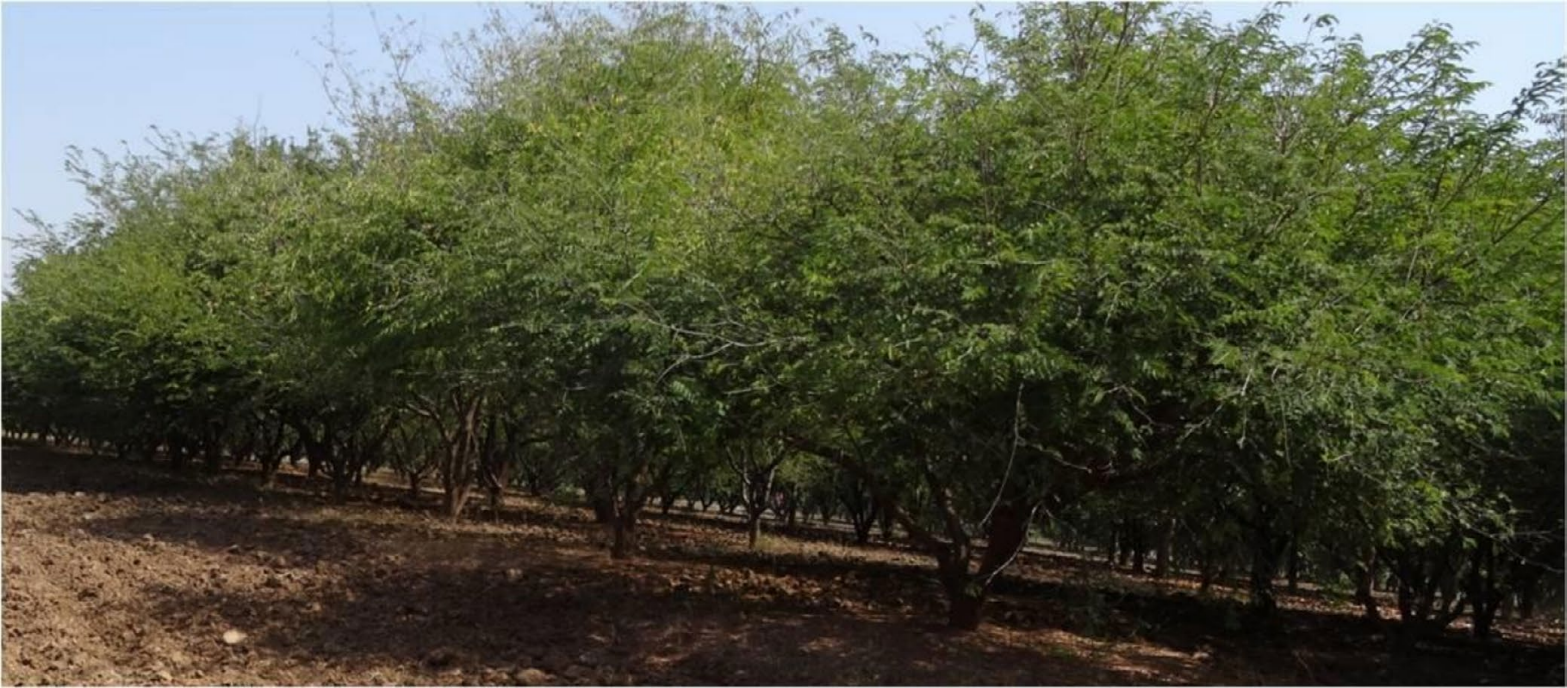
View of the experimental field of tamarind cultivars.

The study was carried out at the ICAR‐CIAH Central Horticultural Experiment Station (CHES), Vejalpur, Panchmahal, Gujarat, India (73°33′ E, 22°41′ N) with an altitude of 113 m above sea level for four seasons from 2020 to 2024. The experimental site is hot and semi‐arid, and its annual rainfall is 750–850 mm. Its soil has a clay‐loam to clay texture with a rocky and calcareous base. Its organic matter content is 0.45%–0.73% and its pH is 6.5. A total of 10 tamarind cultivars, propagated by vegetative means of softwood grafting, were established in the field in 2008 in a square planting system at 5 m spacing (Table 1, Figure 1).

**TABLE 1:** List of tamarind cultivars used in the study.

No.	Cultivar	Pedigree	Source	Specific characteristic
1	Pratisthan	Selection	FRS, Aurangabad	Sweet pulped and high yielder
2	T‐263	Selection	FRS, Aurangabad	Regular bearer and high yielder
3	Ajanta	Selection	FRS, Aurangabad	Regular bearer
4	DTS‐1	Selection	UAS, Dharwad	Precocious bearer
5	Red Type	Selection	HC and RI, Periyakulam	Pods are bold, lengthy, and fleshy
6	Sweet Type	Selection	CHES, Vejalpur	Sweet pulped and low yielder
7	Vantoor	Landrace	RRS, Aruppukottai	Sweet acidic pulp
8	Urigum	Landrace	RRS, Aruppukottai	Bears 2–3 pods per bunch
9	Goma Prateek	Selection	CHES, Vejalpur	Regular bearer and semi‐dwarf
10	PKM‐1	Selection	HC and RI, Periyakulam	Pods born in clusters of 4–5

**FIGURE 2:**
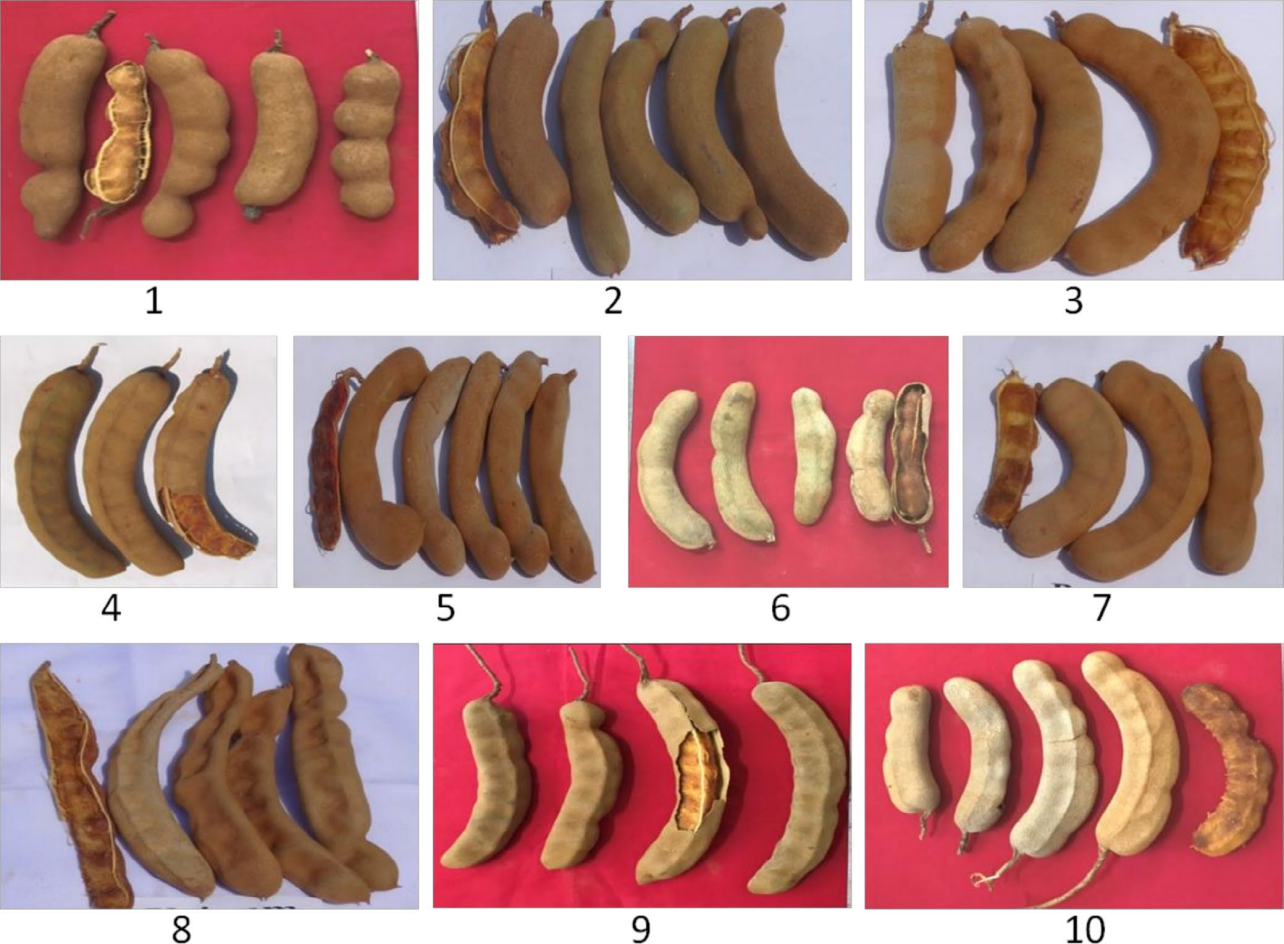
Ten Indian tamarind cultivars studied: (1) Pratisthan, (2) T‐263, (3) Ajanta, (4) DTS‐1, (5) Red Type, (6) Sweet Type, (7) Vantoor, (8) Urigam, (9) Goma Prateek, and (10) PKM‐1.

To maintain tree growth and productivity, the recommended uniform horticultural practices were adopted for the experimental site (Singh and Singh 2013). The outer shell was removed and deseeded after harvesting the tamarind brown ripened pods (Figure 2). Deseeded pulp was used to determine pulp chemical, mineral, and biochemical contents including antioxidant activity.

### 2.2 Tree Growth and Pod Yield Characters

Tree growth and yield attributing characters, including tree height (TH), stem girth (SG), tree spread in north–south (TNS) and east–west (TEW) directions, number of branches/tree (NPB), average number of flowers/inflorescence (NFI), pod yield per tree (PdYT), and pod yield per hectare (PdYH) were meticulously recorded using the standard procedures (Singh et al. 2025). Tree growth related characters were recorded in the second week of March, prior to pod harvesting. Pod yield per tree was calculated based on the total pods harvested during the fruiting period (second week of March to first week of April). Yield per hectare was computed from per‐tree yield and planting density.

### 2.3 Pod Physical Characters

For each variety, 10 commercially mature fruits were harvested from all canopy directions of three randomly selected trees per replication. The pod weight (PdWt) (g), pulp weight (PWt) (g), shell weight (ShWt) (g), fiber weight (FWt) (g), and seed weight (SdWt) (g) were recorded using a precision balance with 0.01 g accuracy. Pod length (PdL) (cm) was measured with the help of a measuring tape from the distance between the pod tip and the pedicel. Pod breadth (PdB) (cm) was recorded using a Vernier caliper (Mitutoyo, Japan), ensuring precise measurements. The number of seeds/pods (NSP) was visually counted. Pulp percent (PP) was determined as pulp weight/pod weight × 100. The real pulp value (RPV) was calculated as pulp percent × (pulp weight/100). Pulp to seed ratio (PSR) was calculated as seed weight/pulp weight.

### 2.4 Pulp Quality Traits and Mineral Contents

The clarified pulp extract was used to estimate total soluble solids (°Brix) and titratable acidity (%). For Total soluble solids (TSS) analysis, pulp extracts were diluted with distilled water (1:9, w/v), and the observed °Brix reading was corrected using the appropriate dilution factor (×10) to estimate the original TSS. Measurements were performed at room temperature using an Erma hand refractometer (0–32 °Brix). Titratable acidity, expressed as percent tartaric acid equivalent, was quantified by titrating against N/10 NaOH using phenolphthalein as the endpoint indicator according to AOAC (2023). Ascorbic acid content (mg 100 g^−1^) was determined using the 2,6‐dichlorophenolindophenol dye titration method following AOAC (2023). Total sugars (%) were estimated by the Lane and Eynon method using Fehling's solution according to the procedure described in AOAC (2023). The sweetness of the pulp (TSA) was calculated as the ratio between sugars and tartaric acid (Van den Bilcke et al. 2014). The mineral contents of calcium (Ca), phosphorus (P), potassium (K), and iron (Fe) in pod pulp were determined using diacid‐digested samples following Mishra et al. (2022). One gram of pulp was digested in 25 mL of a diacid mixture (concentrated nitric acid and perchloric acid in a 9:4 ratio) until a clear solution was obtained, and the final volume was adjusted to 100 mL with distilled water. Calcium was quantified by EDTA titration according to Bhargava and Raghupathi (1993). Phosphorus was estimated colorimetrically using a spectrophotometer (ELICO SL 164, India) following the standard molybdate method described by Bhargava and Raghupathi (1993). Potassium was determined using a flame photometer (ELICO CL 361, India) as per the same procedure. Iron content was measured by the ortho‐phenanthroline method at 515 nm using a spectrophotometer (ELICO SL 164, India) following Jeffery et al. (1989).

### 2.5 Biochemical and Antioxidants

Total phenolic content of pulp extracts was determined using the Folin–Ciocalteu method following Berwal et al. (2021) and expressed as mg gallic acid equivalents (GAE) per 100 g fresh weight (FW). Total flavonoid content was estimated using the aluminum chloride colorimetric method as described by Medini et al. (2014). Briefly, 1 mL of extract was mixed with 0.3 mL of 10% AlCl_3_, 0.3 mL of 5% NaNO_2_, and 3.4 mL of 1 M NaOH. The reaction mixture was incubated at room temperature for 15 min, and absorbance was recorded at 510 nm against a reagent blank (Berwal et al. 2021). Total flavonoid content was expressed as mg catechol equivalents (CE) per 100 g FW. Antioxidant activity of the pulp extract was assessed using the DPPH radical scavenging assay with slight modifications (Berwal et al. 2016). Methanolic pulp extracts (100 μL) at concentrations ranging from 100 to 500 μg mL^−1^ were reacted with 2.9 mL of 0.006% methanolic DPPH solution and incubated in the dark for 10 min. Absorbance was measured at 517 nm using a UV–Vis spectrophotometer (UV‐2550; SHIMADZU). A control containing 100 μL distilled water in place of extract was included. Ascorbic acid (10–50 μg) was used as the reference standard, and antioxidant activity was expressed as percent inhibition.

### 2.6 Statistical Analyses

The experiment was conducted in a randomized complete block design (RCBD) with three replications, each consisting of two trees per variety. Tree growth and yield‐related traits were measured from randomly selected trees (*n* = 3) of each tamarind variety. Pod physical, biochemical, and mineral analyses were performed on pooled samples of 10 fully ripened pods per replicate, with three biological replicates (Obulesu and Bhattacharya 2011). Data were subjected to analysis of variance (ANOVA) using SAS Version 9.1 (SAS Institute Inc., Cary, NC, USA) to test for significant differences among varieties. When ANOVA indicated significant effects (*p* ≤ 0.05), Tukey's post hoc test was applied for mean comparison. Multivariate analyses and graphical visualization were conducted in RStudio version 2023.3.0+386 (Posit Team 2023). Heatmaps were generated using appropriate R packages for data visualization. Principal component analysis (PCA), parallel coordinate plots, and cluster analysis were performed using the FactoMineR, factoextra, dendextend, cluster, and ggplot2 packages (Pearson 1901; Sokal and Michener 1958).

## 3 Results

### 3.1 Analysis of Variance and Mean Comparison

Valuable insights were provided into the variability of growth, yield, and quality traits across the 10 tamarind cultivars, highlighting both commercial and nutritional aspects that are important for selection in breeding programs (Tables 2 and 3). The analysis of variance (ANOVA) demonstrated that the variety factor significantly influenced all measured traits (*p* < 0.001), indicating strong variability between cultivars. This variability is essential for identifying superior genotypes for different purposes. The differences in tree height, stem girth, and tree spread provide important information for selecting tamarind cultivars with better vigor and potential for higher yields. For instance, Ajanta and Goma Prateek exhibited superior growth, with tree heights of 6.34 and 6.47 m, respectively, making them ideal for maximizing space and yield in orchards. Significant variations in pod length, width, and weight, as well as pulp weight and percentage, are critical for assessing the quality and marketability of tamarind. Goma Prateek, with the longest pods (15.0 cm) and higher pulp weight, stands out for its higher fruit quality, whereas Pratisthan demonstrated the highest pulp percentage (10.3 g/pod) and real pulp value (5.13), indicating its potential for higher pulp yield and quality (Table 2). The differences in sweetness‐related traits, such as total soluble solids (TSS) and sugar content, are vital for culinary use, where sweetness balance is crucial. Goma Prateek excelled in sweetness and nutritional quality, showcasing higher levels of vitamin C (24.1 mg 100 g^−1^), total phenols (67.9 mg 100 g^−1^), total flavonoids (35.5 mg 100 g^−1^), and antioxidants (89.4%). Moreover, the superior mineral content, especially calcium (0.75%) and iron (0.24%) content, enhances its nutritional profile, making it a promising candidate for health‐conscious markets (Table 3).

**TABLE 2:** Mean comparison of the measured growth and yield traits in tamarind cultivars.

Cultivar	TH	SG	TEW	TNS	NPB	NFI	PdL	PdB	SdWt	NSP	ShWt	FWt	PWt	PSR	PP	RPV	PdWt	PdYT	PdYH
Ajanta	6.47a	93a	6.26a	6.15a	7.83a	12.6a	12.4abc	2.25bc	5.90a	6.66a	4.93ab	1.06a	6.73abcd	0.88a	36.6c	2.47 cd	18.6ab	17.3ab	6.94ab
DTS‐1	5.71ab	75.2bc	5.60abc	5.57ab	6.33a	9.66a	11.8bc	2.02c	4.11a	6.00a	3.27ab	0.96a	5.45d	0.75ab	39.3bc	2.15d	13.7ab	17.8ab	7.13ab
Goma Prateek	6.34a	77.1b	5.94ab	5.83ab	7.60a	13.1a	15.0a	2.65b	5.98a	7.83a	5.05a	0.54b	9.21abc	0.65ab	44.2abc	4.08abc	20.7a	25.1a	10.0a
PKM‐1	5.14bc	72.1bc	4.98bcd	4.89bcd	7.83a	11.6a	10.7c	2.73b	4.75a	6.43a	3.32ab	0.81ab	6.21bcd	0.80a	41.3bc	2.22 cd	15.1ab	14.9ab	5.97ab
Pratisthan	5.07bc	77.1b	5.07bc	5.00bc	7.50a	14.3a	11.3bc	2.51bc	4.98a	6.33a	4.87ab	0.54b	10.3a	0.48b	49.8a	5.13a	20.7a	18.7ab	7.51ab
Red Type	5.83ab	67.3bcd	5.46abc	5.35abc	4.00a	12.3a	13.8ab	3.79a	4.39a	5.66a	4.56ab	0.73ab	7.45abcd	0.59ab	43.2abc	3.24bcd	17.1ab	12.4b	4.98b
Sweet Type	3.64d	67.3bcd	3.80e	4.49cde	7.60a	12.6a	10.6c	2.38bc	4.18a	5.83a	3.26ab	0.44b	5.77 cd	0.72ab	42.5abc	2.45 cd	13.6ab	11.0b	4.42b
T‐263	3.92d	62.1 cd	4.03de	3.94de	10.3a	13.3a	10.5c	2.35bc	5.06a	8.66a	4.18ab	0.76ab	7.00abcd	0.72ab	41.0bc	2.88bcd	17.0ab	9.21b	3.68b
Urigam	4.45 cd	67.5bcd	4.65cde	3.98de	5.33a	11.2a	11.1bc	2.65b	3.34a	6.13a	2.85b	0.48b	4.94d	0.69ab	42.6abc	2.06d	11.6b	12.1b	4.85b
Vantoor	4.11d	54.1d	3.71e	3.81e	5.76a	10.8a	10.1c	2.48bc	5.55a	5.83a	4.79ab	0.72ab	9.48ab	0.58ab	46.2ab	4.37ab	20.5a	10.1b	4.06b
*F*‐value	33.1	14.89	19.43	17.55	0.443	0.761	6.434	14.69	1.972	0.7	3.774	7.003	6.53	3.287	5.532	8.494	4.292	4.033	4.039
*p*	< 0.001	< 0.001	< 0.001	< 0.001	0.895	0.652	< 0.001	< 0.001	0.098	0.702	0.006	< 0.001	< 0.001	0.012	< 0.001	< 0.001	0.003	0.004	0.004

*Note:* The differences between the means indicated by different letters in the same column are significant at the *p* < 0.001 level.

Abbreviations: FWt (g), fiber weight; NFI, number, number of flowers/inflorescence; NPB, number, number of primary branches/tree; NSP (number), number of seeds/pod; PdB (cm), pod breadth; PdL (cm), pod length; PdWt (g), pod weight; PdYH (t), pod yield ha^−1^; PdYT (kg), pod yield tree^−1^; PP (%), pulp percentage; PSR, pulp:seed; PWt (g), pulp weight; RPV, real pulp value; SdWt (g), seed weight; SG (cm), stem girth; ShWt (g), shell weight; TEW (m), tree spread–east–west; TH (m), tree height; TNS (m), tree spread–north–south.

**TABLE 3:** Mean comparison of the measured quality traits in tamarind cultivars.

Cultivar	TSS	TA	TS	RS	NRS	TSA	VC	TP	TF	AA	K	P	Ca	Mg	Na	Fe
Ajanta	59.4abc	11.7abc	46.5abc	36.4a	9.60de	3.98bcde	14.9d	61.5a	34.5ab	75.0b	0.78b	0.21e	0.36b	0.36abcd	0.17abc	0.13bc
DTS‐1	59.5abc	11.5abc	45.4abcd	35.7a	9.21e	3.91cde	19.6c	39.9c	19.4c	52.8c	0.74b	0.34b	0.81a	0.47a	0.24ab	0.11bc
Goma Prateek	64.4a	13.6a	48.3a	37.7a	10.0cde	3.63def	24.1a	67.9a	35.5a	89.4a	0.96b	0.29bcde	0.75a	0.42abc	0.09c	0.24a
PKM‐1	60.8ab	12.4ab	48.1a	37.6a	9.98cde	3.57ef	14.3d	59.4a	31.9ab	75.3b	0.80b	0.29bcde	0.28b	0.40abcd	0.15abc	0.12bc
Pratisthan	62.9a	11.7abc	48.1a	31.9b	15.3a	4.21bc	11.1f	43.1c	26.8bc	54.8c	1.48a	0.53a	0.78a	0.45ab	0.16abc	0.15bc
Red Type	56.1bc	9.99 cd	44.7abcd	37.5a	6.86f	4.06bcd	21.9b	57.7ab	31.9ab	70.8b	1.07b	0.23cde	0.30b	0.38abcd	0.08c	0.18abc
Sweet Type	59.3abc	8.07d	43.8bcd	36.6a	6.84f	5.62a	20.2c	48.9bc	22.7c	57.8c	1.51a	0.30bcd	0.73a	0.41abc	0.26a	0.13bc
T‐263	53.1c	12.7ab	42.2d	30.8b	10.8bc	3.38f	12.7e	45.4c	21.5c	51.7c	0.96b	0.31bc	0.44b	0.33bcd	0.18abc	0.12bc
Urigam	65.1a	11.0bc	47.8ab	36.8a	10.5 cd	4.35bc	20.3c	63.4a	35.7a	77.3b	0.77b	0.22de	0.40b	0.32 cd	0.14bc	0.10c
Vantoor	60.3ab	9.94 cd	43.3 cd	31.0b	11.7b	4.41b	19.6c	62.0a	34.8a	76.6b	0.74b	0.24cde	0.36b	0.28d	0.16abc	0.20ab
*F*‐value	7.478	14.35	7.398	16.51	122.5	51.19	302.7	22.91	15.92	62.39	15.88	23.98	23	5.668	5.984	7.339
*p*	< 0.001	< 0.001	< 0.001	< 0.001	< 0.001	< 0.001	< 0.001	< 0.001	< 0.001	< 0.001	< 0.001	< 0.001	< 0.001	< 0.001	< 0.001	< 0.001

*Note:* The differences between the means indicated by different letters in the same column are significant at the *p* < 0.001 level.

Abbreviations: AA (%), antioxidant activity; Ca (%), calcium; Fe (%), iron; K (%), potassium; Mg (%), magnesium; Na (%), sodium; NRS (%), non‐reducing sugars; P (%), phosphorus; RS (%), reducing sugars; TA (%), tartaric acid; TF (CE mg 100 g^−1^), total flavonoids; TP (GAE mg 100 g^−1^), total phenols; TS (%), total sugars; TSA, sweetness; TSS (^o^Brix), total soluble solids; VC (mg 100 g^−1^), vitamin C.

### 3.2 Character Association Studies

**FIGURE 3:**
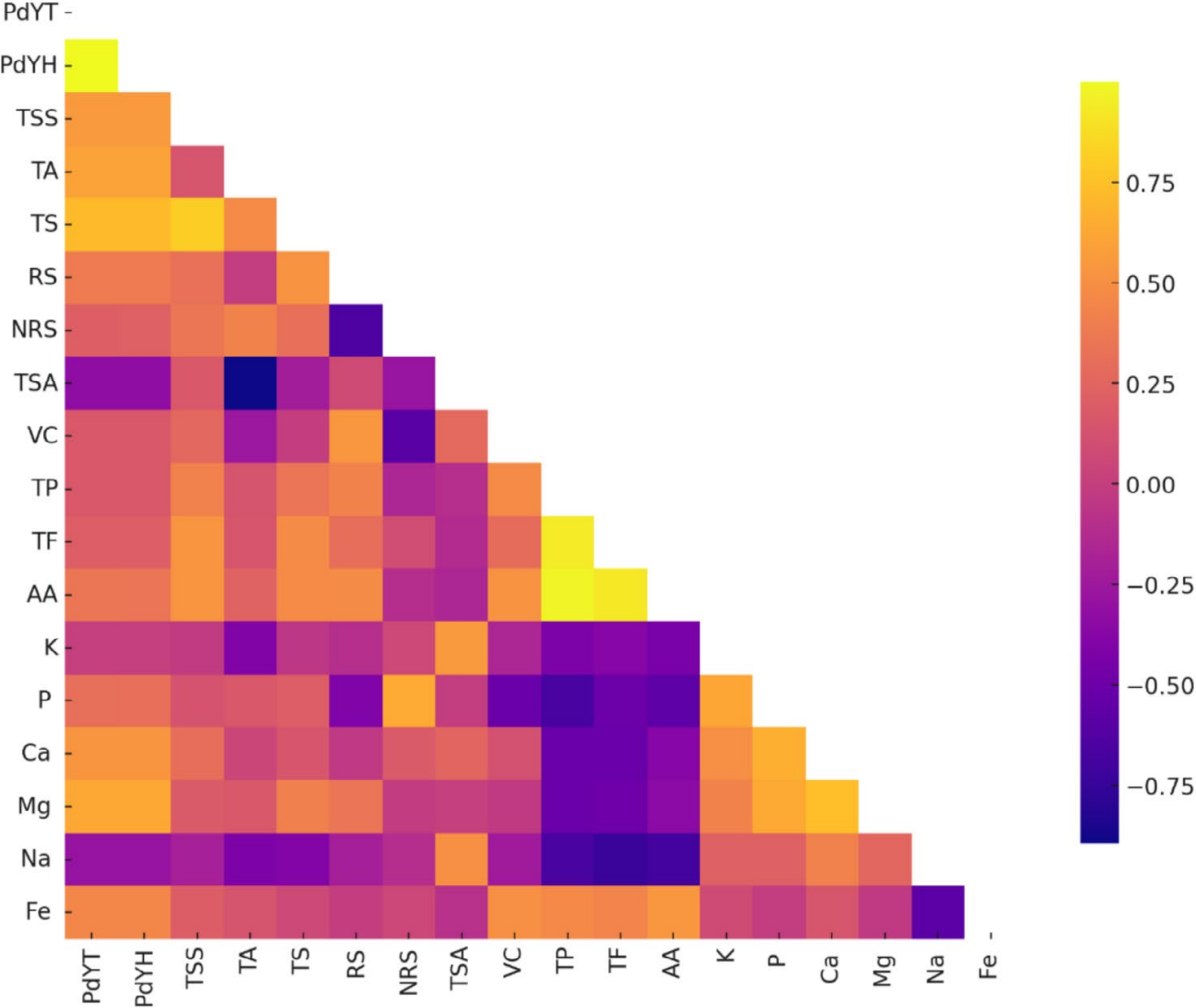
Genotypic correlations between morphological, biochemical, and mineral traits of tamarind cultivars. Heatmap illustrating genotypic correlation coefficients (*r*_g_) among growth, yield, pulp quality, antioxidant, and mineral traits. Color intensity represents the magnitude and direction of correlation, with yellow to orange indicating strong positive correlations and purple to dark blue indicating strong negative correlations. The lower triangular matrix is displayed for clarity. Traits include pod yield per tree (PdYT), pod yield per hectare (PdYH), total soluble solids (TSS), titratable acidity (TA), total sugars (TS), reducing sugars (RS), non‐reducing sugars (NRS), sweetness index (TSA), vitamin C (VC), total phenolics (TP), total flavonoids (TF), antioxidant activity (AA), potassium (K), phosphorus (P), calcium (Ca), magnesium (Mg), sodium (Na), and iron (Fe).

**FIGURE 4:**
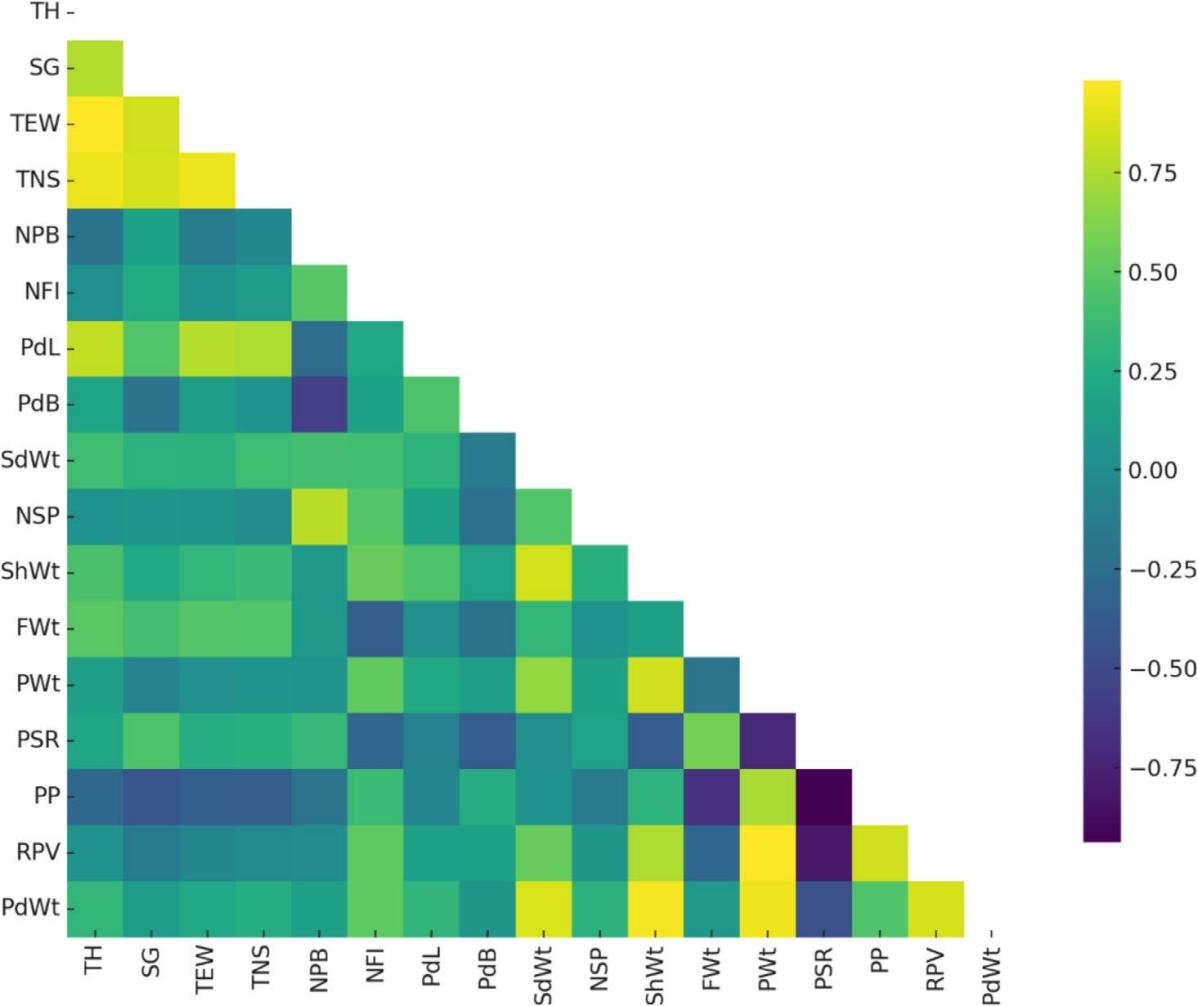
Phenotypic correlations between morphological, biochemical, and mineral traits of tamarind cultivars. Heatmap representing phenotypic correlation coefficients (*r*_p_) among tree growth, yield, fruit physical, pulp quality, antioxidant, and mineral traits. Color gradients indicate the strength and direction of correlations, with yellow to green representing strong positive associations and blue to purple indicating negative associations. Only the lower triangular matrix is shown to enhance clarity. Traits include tree height (TH), stem girth (SG), tree spread east–west (TEW), tree spread north–south (TNS), number of primary branches (NPB), number of flowers per inflorescence (NFI), pod length (PdL), pod breadth (PdB), seed weight (SdWt), number of seeds per pod (NSP), shell weight (ShWt), fiber weight (FWt), pulp weight (PWt), pulp‐to‐seed ratio (PSR), pulp percent (PP), real pulp value (RPV), and pod weight (PdWt).

Phenotypic and genotypic correlation coefficients were analyzed for the traits measured across 10 tamarind cultivars (Figures 3 and 4). The data showed that phenotypic correlation coefficients were generally lower than genotypic ones, highlighting a robust inherent association between the traits.

#### 3.2.1 Tree Growth and Agronomic Characters

Tree growth traits (TH, SG, TEW, and TNS) were strongly and positively correlated among themselves. Pod length (PdL) showed significant positive correlations with TEW (*r*_g_ = 0.768; *r*_p_ = 0.562), TNS (*r*_g_ = 0.744; *r*_p_ = 0.539), pod yield per tree (PdYT; *r*_g_ = 0.702; *r*_p_ = 0.623), and pod yield per hectare (PdYH; *r*_g_ = 0.701; *r*_p_ = 0.624). Pulp weight (PWt), a key economic trait, exhibited very strong positive correlations with real pulp value (RPV; *r*_g_ = 0.983; *r*_p_ = 0.976), pod weight (PdWt; *r*_g_ = 0.938; *r*_p_ = 0.932), shell weight (ShWt; *r*_g_ = 0.846; *r*_p_ = 0.801), pulp percent (PP; *r*_g_ = 0.735; *r*_p_ = 0.617), and iron content (Fe; *r*_g_ = 0.755; *r*_p_ = 0.570). RPV was likewise strongly associated with PP (*r*_g_ = 0.841; *r*_p_ = 0.767). Seed weight (SdWt) showed strong positive correlations with PdWt (*r*_g_ = 0.871; *r*_p_ = 0.851) and ShWt (*r*_g_ = 0.850; *r*_p_ = 0.700), but a negative association with sweetness index (TSA; *r*_g_ = −0.368; *r*_p_ = −0.202). Pulp‐to‐seed ratio (PSR) exhibited strong negative correlations with PP (*r*_g_ = −0.938; *r*_p_ = −0.899) and RPV (*r*_g_ = −0.810; *r*_p_ = −0.662), whereas fiber weight (FWt) was negatively correlated with PP (*r*_g_ = −0.675; *r*_p_ = −0.531). Pulp percent (PP) showed positive correlations with phosphorus (P; *r*_g_ = 0.595; *r*_p_ = 0.560), potassium (K; *r*_g_ = 0.514; *r*_p_ = 0.375), and non‐reducing sugars (NRS; *r*_g_ = 0.589; *r*_p_ = 0.490). Overall, pulp‐related traits were strongly interlinked and positively associated with key mineral elements.

#### 3.2.2 Biochemical and Mineral Characters

Total soluble solids (TSS) exhibited strong positive correlations with total sugars (TS; *r*_g_ = 0.804; *r*_p_ = 0.764), ascorbic acid (AA; *r*_g_ = 0.526; *r*_p_ = 0.428), total flavonoids (TF; *r*_g_ = 0.522; *r*_p_ = 0.471), and total phenolics (TP; *r*_g_ = 0.418; *r*_p_ = 0.375). Titratable acidity (TA) showed a strong negative correlation with sweetness index (TSA; *r*_g_ = −0.894; *r*_p_ = −0.804). Reducing sugars (RS) were positively associated with vitamin C (*r*_g_ = 0.539; *r*_p_ = 0.505) and antioxidant traits, but negatively correlated with non‐reducing sugars (NRS; *r*_g_ = −0.651; *r*_p_ = −0.583). Among minerals, potassium (K), phosphorus (P), and calcium (Ca) were positively interrelated. Iron (Fe) exhibited a positive correlation with antioxidant activity (*r*_g_ = 0.541; *r*_p_ = 0.459) and a negative correlation with sodium (Na; *r*_g_ = −0.580; *r*_p_ = −0.418). In general, sodium showed negative associations with most quality and antioxidant traits.

### 3.3 PCA and Biplot

PCA was employed to eliminate redundancy in the data set. The percentage variation (98.90%) related to the first eight principal components (PCs) and the vector loadings for each trait and PC is shown in Table 4 and Table S1. A large part of the contribution in distinguishing genotypes was attributed to traits with positive and negative values. The 27.40% of the variance was explained by PC1, as the most significant component, distinguishing genotypes based on morphological traits such as pod yield/tree (PdYT), pod yield/ha (PdYH), tree height (TH), and pod length (PdL). These variables showed strong negative loadings, indicating an inverse relationship with PC1. The PC2 accounted for 20.60% of the total variance and included the pulp percentage (PP), real pulp value (RPV), pulp weight (PWt), and phosphorus (P) with high positive values and also reducing sugar (RS) and pulp seed ratio (PSR) with high negative values. The PC3 represented 16.35% of the total variance and included total flavonoids (TF), total phenols (TP), antioxidants (AA), and vitamin C (VC), called antioxidant‐related traits. The mineral content of the pulp was correlated with PC4, whereas non‐reducing sugars (NRS) and total sugars (TS) were correlated with PC5.

**TABLE 4:** Principal component analysis for quantitative and qualitative traits of tamarind cultivars.

Principal component	Eigenvalue	% Of total variance	Cumulative variance (%)
PC1	3.09	27.40	27.40
PC2	2.68	20.60	48.00
PC3	2.39	16.35	64.35
PC4	2.12	12.81	77.16
PC5	1.60	07.34	84.50
PC6	1.34	05.09	89.59
PC7	1.32	04.99	94.58
PC8	1.23	04.32	98.90

**FIGURE 5:**
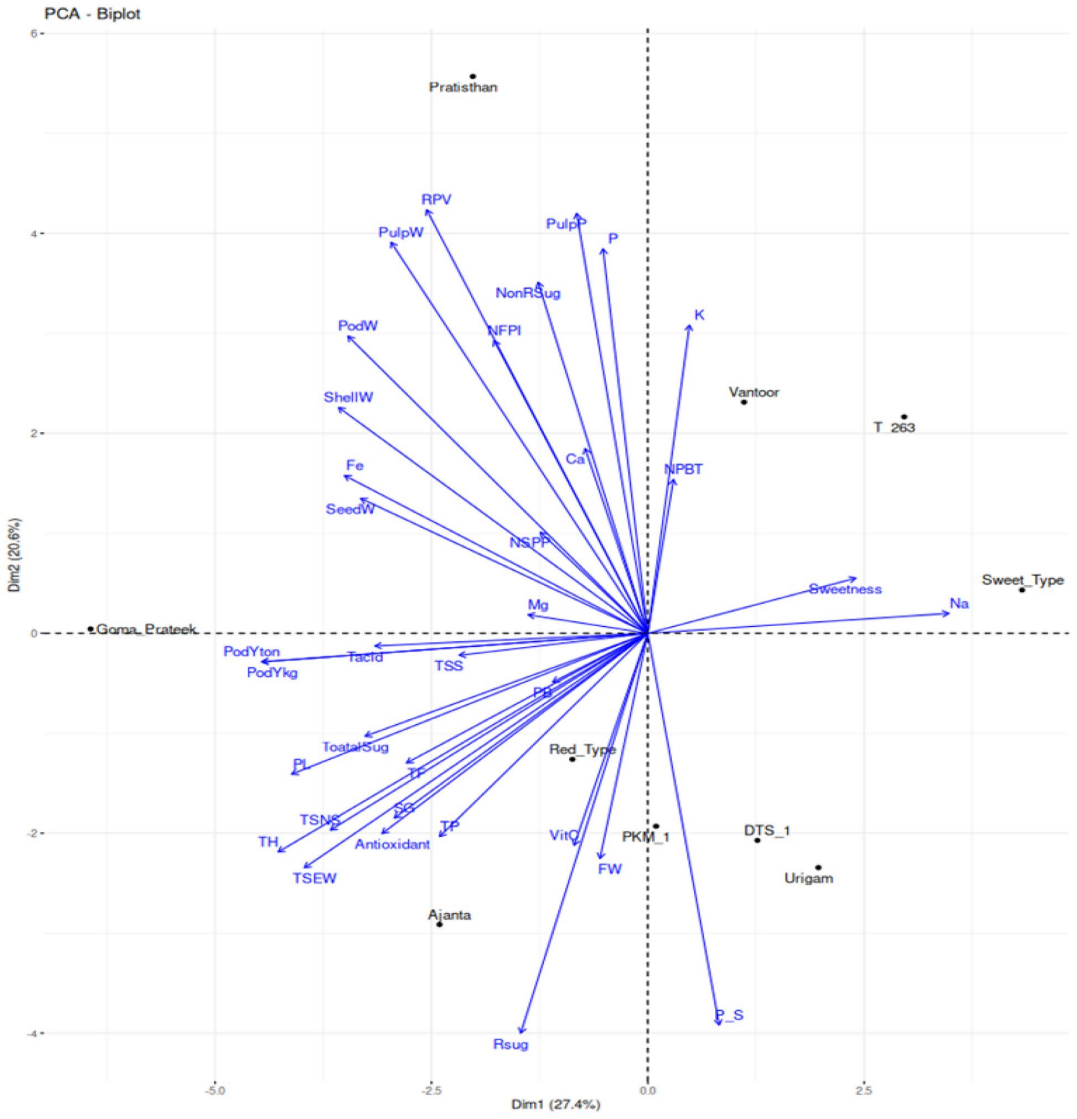
PCA biplot showing distribution and trait associations of 10 tamarind cultivars. Principal component analysis (PCA) biplot illustrating the distribution of cultivars and the contribution of morphological, yield, biochemical, and mineral traits along the first two principal components (PC1 = 27.40% and PC2 = 20.60% of total variance). Vectors represent trait loadings, with arrow length indicating the magnitude of contribution and direction indicating correlation structure. Cultivar positions reflect their relative similarity and divergence based on combined trait performance.

The PCA biplot (PC1 vs. PC2; Figure 5) explained 57.22% of the total variance and revealed clear dispersion of cultivars across all four quadrants, indicating substantial phenotypic divergence. Cultivars positioned in the negative PC1 region were associated with superior yield‐related traits, whereas those in the positive PC2 region were characterized by higher pulp content and phosphorus levels. Traits such as RPV, pulp percentage, Na, reducing sugars, and tree height emerged as key contributors to genotype differentiation and may serve as important selection criteria for breeding and value‐addition programs.

### 3.4 Cluster Analysis

**FIGURE 6:**
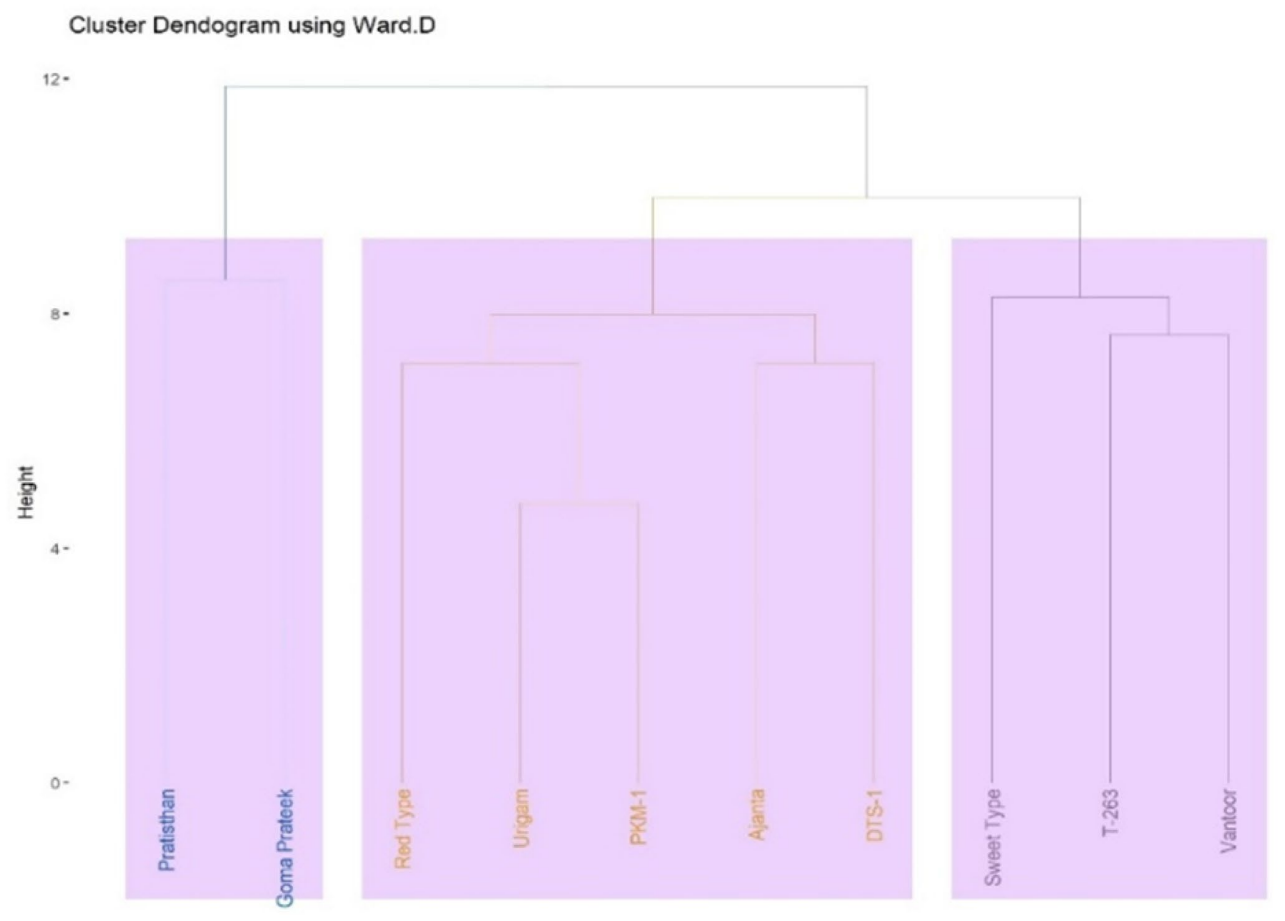
Dendrogram of 10 tamarind cultivars created using the average distance between cluster analyses based on 35 quantitative traits. Dendrogram generated using Ward's method and Euclidean distance to illustrate genetic divergence among cultivars. The vertical axis represents linkage distance (cluster height), whereas the horizontal grouping shows similarity relationships. The analysis grouped the cultivars into three major clusters, indicating substantial variability and distinct trait‐based divergence among the evaluated genotypes.

Cluster analysis is an important tool for the selection of superior and contrasting genotypes that can be candidates as parents for tamarind breeding programs. The dendrogram showed the relative magnitude of resemblance among the cultivars in different clusters (Figure 6). The 10 tamarind cultivars clustered into three major groups (cluster I, II, and III) based on 35 measurable traits (Table 5). Cluster I consisted of Pratisthan and Goma Prateek. Cluster II was further divided into sub‐clusters IIA and IIB. Sub‐cluster IIA included Red Type, Urigam, and PKM 1, whereas sub‐cluster IIB comprised Ajanta and DTS 1. Cluster III was also further divided into sub‐clusters IIIA and IIIB. Sub‐cluster IIIA included Sweet Type, and sub‐cluster IIIB consisted of T‐263 and Vantoor cultivars.

**TABLE 5:** Cluster composition based on *D* ^2^ statistics of 10 tamarind cultivars in three clusters.

Cluster	Number of germplasm	Name of germplasm
Cluster I	2	Pratisthan, Goma Prateek
Cluster II	3	T‐263, Sweet Type, Vantoor
Cluster III	5	Ajanta, DTS‐1, Red Type, Urigam, PKM‐1

#### 3.4.1 Cluster Mean Analysis

Clusters mean differences are quite distinct, which shows wider variation among the various traits studied (Table 6). Cluster I exhibited the highest mean values for tree growth characters (TH, SG, TEW, TNS), yield attributes (PdL, PdB, ShWt, PWt, PP, RPV, PdWt), pod yield per tree (PdYT), and pod yield per hectare (PdYH), as well as chemical quality attributes (TSS, TA, TS, NRS), bioactive compounds (TF, AA), and mineral contents (K, P, Ca, Mg, Fe). Cluster II displayed lower mean values for most growth, yield, and chemical quality traits compared to Cluster I, with some exceptions like Na and TSA, which were higher in Cluster II. Cluster III showed higher mean values for FWt, PSR, RS, VC, TP, and TF, but lower values for other traits including pod weight and mineral contents like K and Ca.

**TABLE 6:** Cluster mean analysis for 35 traits in 10 tamarind cultivars.

Sr. no.	Character	Clusters I	Clusters II	Clusters III
1	Tree height (TH)	5.71	3.89	5.52
2	Stem girth (SG)	76.95	61.07	75.02
3	Tree spread‐East–West (TEW)	5.51	3.85	5.39
4	Tree spread‐North–South (TNS)	5.42	4.08	5.19
5	Number of primary branches/tree (NPB)	7.55	7.90	6.24
6	Number of flower/inflorescence (NFI)	13.75	12.29	11.51
7	Pod length (PdL)	13.21	10.48	12.04
8	Pod breadth (PdB)	2.58	2.40	2.69
9	Seed weight (SdWt)	5.50	4.97	4.50
10	Number of seeds/pod (NSP)	7.05	6.77	6.18
11	Shell weight (ShWt)	4.96	4.08	3.79
12	Fiber weight (FWt)	0.55	0.64	0.81
13	Pulp weight (PWt)	9.76	7.42	6.16
14	Pulp: seed (PSR)	0.57	0.67	0.74
15	Pulp percentage (PP)	47.01	43.28	40.63
16	Real pulp value (RPV)	4.61	3.24	2.50
17	Pod weight (PdWt)	20.75	17.07	15.26
18	Pod yield/tree (PdYT)	21.97	10.15	14.94
19	Pod yield/ha (PdYH)	8.78	4.06	5.98
20	Total soluble solids (TSS)	63.70	57.63	60.23
21	Tartaric acid (TA)	12.67	10.24	11.36
22	Total sugars (TS)	48.20	43.13	46.56
23	Reducing sugars (RS)	34.87	32.82	36.84
24	Non‐reducing sugars (NRS)	12.69	9.81	9.24
25	Sweetness (TSA)	3.92	4.48	3.98
26	Vitamin C (VC)	17.70	17.50	18.26
27	Total phenols (TP)	55.54	52.15	56.45
28	Total flavonoids (TF)	31.19	26.37	30.67
29	Antioxidant activity (AA)	72.15	62.07	70.26
30	Potassium (K)	1.22	1.07	0.84
31	Phosphorus (P)	0.42	0.29	0.26
32	Calcium (Ca)	0.77	0.51	0.44
33	Magnesium (Mg)	0.44	0.35	0.39
34	Sodium (Na)	0.13	0.20	0.16
35	Iron (Fe)	0.20	0.15	0.13

#### 3.4.2 Heat Map Analysis

**FIGURE 7:**
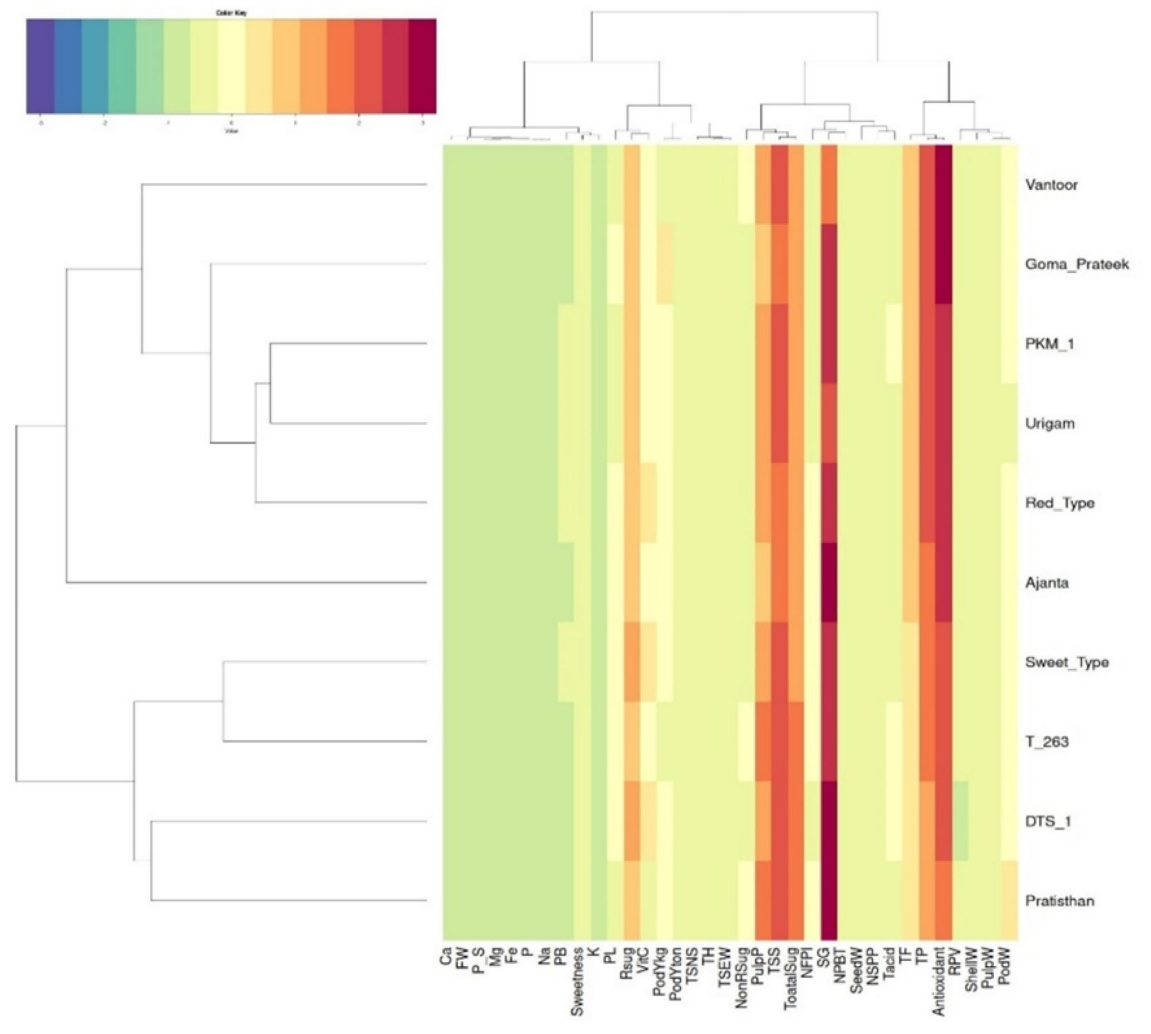
Clustering Heat map of biometric traits of tamarind cultivars. Heatmap showing standardized trait values of 10 tamarind cultivars clustered based on Euclidean distance and hierarchical agglomerative clustering. Color gradients represent relative trait intensity, with cooler colors indicating lower values and warmer colors indicating higher values.

Heat map enabled a clear depiction of the variability of analyzed characters among the cultivars. For example, Goma Prateek emerged as the richest source of antioxidants, total phenols, and total flavonoids, which was visible in a heat map (Figure 7). The two‐way hierarchical clustering and heat map were instrumental in identifying the best variety with optimal quality characteristics.

#### 3.4.3 Intra‐ and Inter‐ Cluster *D* ^2^ Value

**FIGURE 8:**
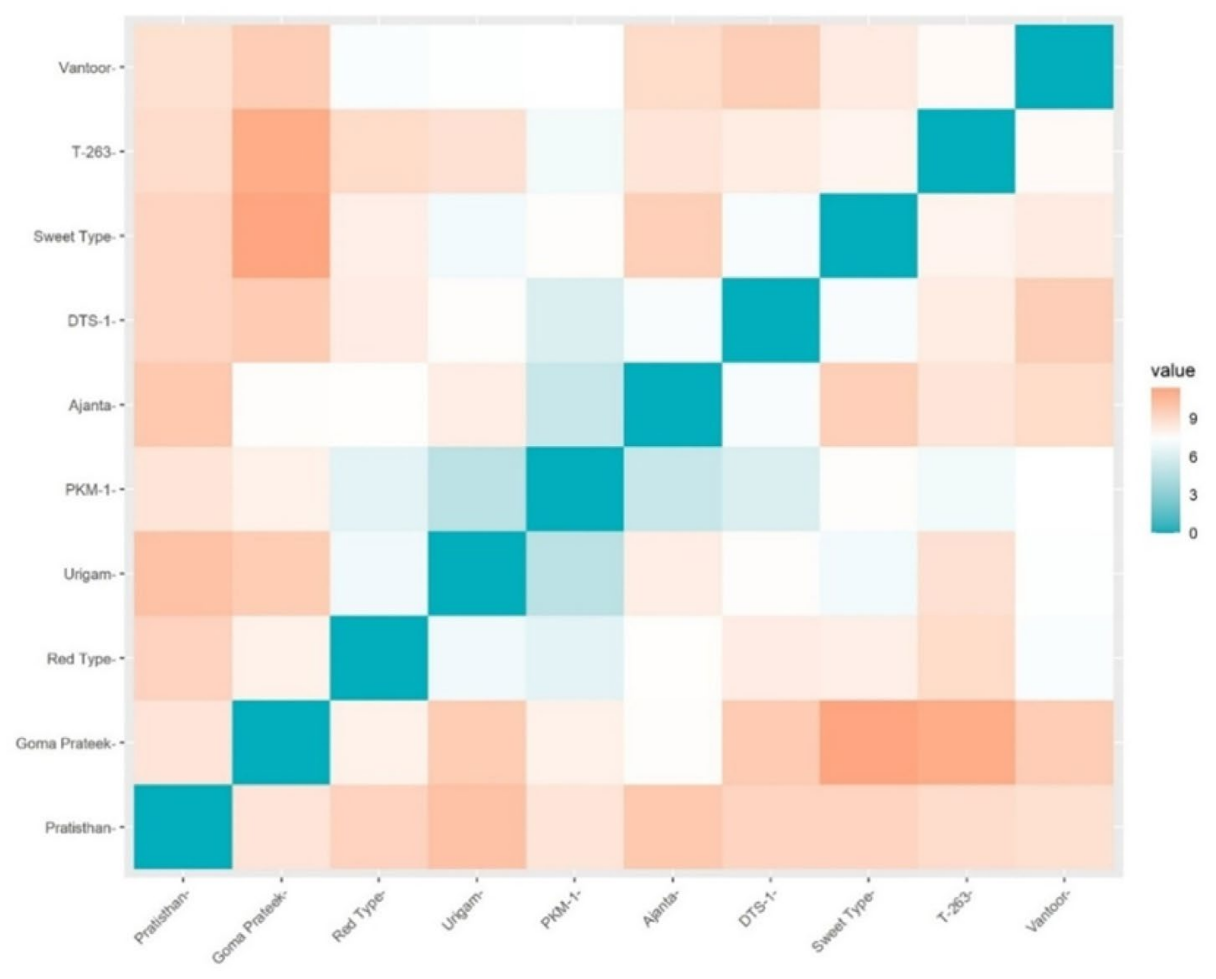
Heat map for genetics distance among cultivars.

The cultivars studied were analyzed for diversity using quantitative and qualitative data with the help of the Ward *D* method (Table 7). The range of intra‐cluster distance was from 36.35 to 47.25, whereas the range of inter‐cluster distance was from 46.57 to 55.40. The highest value (55.40) for inter‐cluster distance was recorded between clusters I and II, whereas it was the lowest (46.57) between clusters II and II. The high range intra‐cluster distance showed the presence of a high range of diversity within a cluster. A maximum cluster distance of 55.40 observed between clusters I and II indicated that cultivars falling in these clusters can be used in the hybridization programs to get better recombinants in the segregating generations. The genetic distance of each genotype, derived using quantitative and qualitative data, revealed that Sweet Type and Goma Prateek have the most divergent genetic distance (11.37) (Table 8 and Figure 8). According to the cluster diagram, Sweet Type and Goma Prateek are grouped into cluster III and cluster I, respectively. Pratisthan and Urigam are grouped into cluster I and cluster II, respectively. The genetic distance between Urigam and PKM‐1 was the smallest (4.77). The cluster analysis indicates that these genotypes viz., Ajanta, Urigam, DTS‐1, and PKM‐1 have the least genetic distance and are grouped into the same cluster (cluster II).

**TABLE 7:** Average Intra and inter‐cluster distance (*D* ^2^) values clusters of tamarind.

Cluster	Cluster I	Cluster II	Cluster III
Cluster I	47.25		
Cluster II	55.40	36.35	
Cluster III	51.38	46.57	39.80

**TABLE 8:** Genetic distances between 10 tamarind cultivars.

Cultivars	Pratisthan	T‐263	Ajanta	DTS‐1	Red Type	Sweet Type	Vantoor	Urigam	Goma Prateek	PKM‐1
Pratisthan	0.00									
T‐263	8.99	0.00								
Ajanta	9.83	8.63	0.00							
DTS‐1	9.31	8.26	7.16	0.00						
Red Type	9.38	9.04	7.49	8.22	0.00					
Sweet Type	9.36	7.94	9.59	7.19	8.13	0.00				
Vantoor	8.78	7.64	9.05	9.61	7.23	8.31	0.00			
Urigam	10.16	8.76	8.21	7.53	6.91	6.96	7.35	0.00		
Goma Prateek	8.57	11.06	7.53	9.76	8.03	11.37	9.66	9.72	0.00	
PKM‐1	8.62	6.94	5.19	5.98	6.35	7.52	7.39	4.77	8.03	0.00

## 4 Discussion

### 4.1 Analysis of Variance and Mean Comparison

To the rational use of tamarind genetic resources in crop breeding and improvement, precise evaluation of genetic and phenotypic diversity is vital. The present study characterized 10 tamarind cultivars using agronomic and biochemical traits to evaluate the extent of genotypic and phenotypic variability, recognize the genetic and morphological relationship, and determine the promising types for further breeding programs. Our study exhibited that the *F*‐values are highly significant (*p* < 0.001) except for agronomic traits such as NPB (*F* = 0.443, *p* = 0.895), NFI (*F* = 0.761, *p* = 0.652), SdWt (*F* = 1.972, *p* = 0.0989), and NSP (*F* = 0.70, *p* = 0.702) which indicates as narrow variability. Particularly noteworthy are the relationships involving pod weight (PWt), which is crucial from an economic standpoint (Mishra et al. 2025). The strong positive association of real pulp value (RPV) with pulp weight (PWt), pod weight (PdWt), and pulp percent (PP) highlights its relevance as an integrated indicator of effective edible yield. Since RPV combines pulp proportion and pulp mass, it provides a more comprehensive assessment of productive efficiency than individual traits alone. Its close linkage with key yield components supports its potential utility as a composite selection index in tamarind improvement programs, in agreement with earlier studies emphasizing pulp‐based indices for cultivar evaluation (Singh and Nandini 2014; Kanupriya et al. 2025; Kumar et al. 2023). Previous studies reported significant variations in tree growth and morphological and quality traits in India (Kanupriya et al. 2025; Mayavel et al. 2025) and different parts of the globe (Adeola and Aworh 2012; Okello et al. 2017). Raut et al. (2022) assessed yield and quality traits including total sugars and tartaric acid, in 22 tamarind accessions from Maharashtra, similar to the present study. Variety Goma Prateek was found to be superior for commercially important traits such as pod length, pulp percent, RPV, TSS (°Brix), tartaric acid (%), non‐reducing sugar (%), reducing sugar (%), total sugar (%), total phenols (mg 100 g^−1^), vitamin C (mg 100 g^−1^), total flavonoids (mg 100 g^−1^), antioxidant (% inhibition), Ca (%), and Fe (%). This analysis's conclusions ought to guide a breeding program that strikes a balance between yield, quality, and nutritional value. A strong genetic relationship that can be benefited from through selective breeding is indicated by the strong positive correlations. Nonetheless, it is imperative to acknowledge the adverse associations to prevent jeopardizing crucial characteristics.

### 4.2 Character Association Studies

Genotypic correlation coefficients were consistently higher than phenotypic correlations, indicating a robust inherent association between the traits. This suggests that these traits should be prioritized as crucial selection criteria for improving PdYT. Furthermore, our study revealed higher genotypic correlations between trait pairs compared with their corresponding phenotypic correlations, indicating a strong genetic association between variables, albeit moderated by environmental influences on their phenotypic expression (Pooja et al. 2018; Raut et al. 2022). TH, SG, TEW, and TNS direction, and TS, PdL, Mg, Ca, TA, TSS, and Fe emerged as the most crucial traits contributing significantly to higher PdYT based on correlation coefficients. Singh and Nandini (2014) reported positive correlations of pulp weight with pod length, epicarp weight, and fiber weight. Similar findings on yield and quality traits in tamarind were also reported by Mayavel et al. (2018) and Pooja et al. (2018). The correlation between yield and quality traits was extensively studied in different tamarind cultivars. Some variables, such as pod height (TH), seed weight (SG), total edible weight (TEW), and total number of seeds (TNS), have substantial positive connections with one another, which suggests that these traits may be interdependent (Yadav et al. 2022). This suggests that enhancements in one characteristic will probably have a favorable effect on others. For example, choosing for higher pod weight (PWt) could improve seed weight and total edible weight at the same time, which are important for customer preference and commercial viability (Yadav et al. 2024). Conversely, traits, such as pod length (PdL), pod yield in tons (PdYT), and pod yield per hectare (PdYH) exhibited negative correlations with characters such as pulp percentage (PP), total soluble solids (TSA), and sodium (Na). This suggests a trade‐off where enhancing yield‐related traits may lead to reductions in pulp quality or nutritional content. To ensure that increased yields do not compromise the overall quality of the fruit, this trade‐off should be carefully managed in breeding programs (Rajamanickam 2020; Mamathashree et al. 2021).

### 4.3 PCA and Biplot

Principal component analysis (PCA) effectively reduced trait dimensionality and identified key variables contributing to genotype differentiation. Trait associations observed in PCA may reflect genetic linkage or pleiotropic effects. PCA has been widely applied in germplasm evaluation to understand trait relationships and genetic divergence (Khadivi‐Khub and Anjam 2014; Mishra et al. 2022; Kanupriya et al. 2025). In the present study, the first five principal components explained 90.11% of the total variation, slightly lower than the 98.61% reported by Kanupriya et al. (2025). PC1 (27.40%) was mainly associated with morphological and yield traits including tree height (TH), pod length (PdL), pod yield per tree (PdYT), and pod yield per hectare (PdYH). The negative loadings among these traits indicate possible trade‐offs, suggesting that yield improvement requires balanced selection. PC2 (20.60%) emphasized pulp quality traits such as pulp percentage (PP), real pulp value (RPV), pulp weight (PWt), and phosphorus (P), underscoring their importance in genotype differentiation. Negative loadings for pulp‐to‐seed ratio (PSR) and reducing sugars (RS) suggest a balance between pulp yield and sugar composition.

In the biplot, RPV, PP, PWt, Ca, Fe, total phenols (TP), total flavonoids (TF), and antioxidant activity (AA) were closely associated with cultivars such as Pratishthan, Goma Prateek, Ajanta, and Red Type, indicating their superiority for yield and nutritional quality traits. In contrast, Vantoor, T‐263, and Sweet Type were more associated with sweetness‐related traits and sodium (Na) content, consistent with previous reports (Ayala‐Silva et al. 2013; Kumar et al. 2023; Reddy et al. 2024; Yadav et al. 2022). The clear separation of cultivars across quadrants of the PC1–PC2 biplot demonstrates substantial genotypic divergence. Key discriminating traits included RPV, PP, Na, reducing sugars, and tree height. These findings highlight the importance of integrating yield, pulp quality, and nutritional traits in tamarind breeding programs. Multivariate approaches such as PCA, correlation matrices, dendrograms, and heatmaps enhance selection efficiency and facilitate identification of superior cultivars combining productivity and quality.

### 4.4 Cluster Analysis

Cluster analysis revealed substantial variability among tamarind cultivars based on quantitative traits, grouping them into three major clusters with further sub‐clustering. The clear separation among clusters reflects significant divergence in biometric and quality attributes. Similar clustering patterns based on morphological and quantitative descriptors have been reported in tamarind and other perennial fruit crops (Ayala‐Silva et al. 2013; Khadivi‐Khub and Anjam 2014; Mamathashree et al. 2021; Mishra et al. 2022; Kumar et al. 2023; Kanupriya et al. 2025; Reddy et al. 2024). Pratishthan and Goma Prateek grouped together in Cluster I, indicating close genetic affinity and possible sharing of favorable yield and quality traits. Such clustering provides practical guidance for parent selection, where inter‐cluster hybridization may enhance genetic variability and heterosis, whereas intra‐cluster crosses may stabilize desirable traits (Divakara et al. 2012; Kanupriya et al. 2025). However, morphological characterization alone may underestimate true genetic diversity due to environmental influence and trait plasticity (Ayala‐Silva et al. 2013; Kumar et al. 2023). The higher inter‐cluster distances compared to intra‐cluster distances confirm substantial genetic divergence among cultivars. Notably, Sweet Type and Goma Prateek exhibited maximum divergence, supporting their potential as contrasting parents in breeding programs, consistent with earlier reports (Divakara et al. 2012; Reddy et al. 2024). Heatmap analysis further identified Goma Prateek as a superior source of antioxidants, total phenols, and total flavonoids. Overall, cluster‐based selection, supported by multivariate analyses, offers a robust framework for improving yield, sweetness, and nutritional quality traits in tamarind breeding.

### 4.5 Limitations and Future Perspectives

Although this study provides integrated compositional profiling of 10 tamarind cultivars, certain limitations should be acknowledged. The experiment was conducted at a single semi‐arid location, and genotype × environment interactions were not quantified. Environmental conditions are known to influence organic acid balance, phenolic accumulation, and mineral uptake in tamarind fruits (Van den Bilcke et al. 2014; Kidaha et al. 2019). Additionally, biochemical evaluation was limited to total phenolics, total flavonoids, and DPPH radical scavenging activity. Although these indices provide an estimate of antioxidant potential, they do not identify individual bioactive compounds responsible for specific health effects (Komakech et al. 2019; Akter et al. 2023). Mineral analysis was performed using conventional wet‐digestion methods; more advanced techniques such as ICP‐OES could improve micronutrient precision (Nagar et al. 2022).

Importantly, the orchard was maintained under a traditional 5 × 5 m spacing system. Planting density influences canopy architecture, light interception, and photosynthetic efficiency, thereby affecting yield and biochemical composition (Reddy et al. 2023). Consequently, the yield and quality traits reported here reflect performance under conventional spacing and may differ under high‐density planting (HDP) systems, particularly for compact cultivars.

Future research should include multi‐location trials to assess trait stability, evaluation under HDP systems, LC–MS‐based metabolomic profiling for compound‐specific characterization, ICP‐based mineral quantification, and integration of sensory and processing assessments to enhance industrial and nutritional applicability.

## 5 Conclusions

This study evaluated 10 tamarind cultivars under semi‐arid conditions using integrated morphological, yield, biochemical, and mineral analyses. Significant varietal differences demonstrated substantial phenotypic diversity, offering strong potential for selection and improvement. Pulp‐related traits, particularly pulp weight (PWt), pulp percentage (PP), and real pulp value (RPV), emerged as key indicators of productive efficiency due to their close association with pod weight and mineral attributes. PCA identified yield traits (PdYT, PdYH, TH, PdL) and pulp quality parameters (PP, RPV, PWt, P) as major contributors to genotype differentiation, whereas cluster analysis revealed considerable divergence, with Sweet Type and Goma Prateek showing maximum genetic distance. Goma Prateek, Pratisthan, and Ajanta combined high yield with superior nutritional and antioxidant properties, making them promising candidates for breeding. Overall, the integrated multivariate approach provides a robust framework for selecting cultivars with enhanced yield, pulp quality, and nutritional value for semi‐arid regions.

## Author Contributions

Conceptualization, methodology, data curation, D.S.M., Y.T., and A.K.; writing – original draft preparation, visualization, L.P.Y., and P.R.; analysis of mineral and biochemical characters, V.V.A., K.C., Y.T., and A.K.; writing – review and editing, D.S.M., V.Y., L.P.Y., J.R., V.V.A., K.C., P.R., P.Ka., and P.Ku.; statistical analysis, P.Ka. and P.Ku. All authors have read and agreed to the published version of the manuscript.

## Funding

The authors have nothing to report.

## Ethics Statement

The authors have nothing to report.

## Conflicts of Interest

The authors declare no conflicts of interest.

## Supporting information


**Table S1:** Trait loadings for the first five principal components.

## Data Availability

The data that support the findings of this study are available from the corresponding author upon reasonable request.
