# Supplementary material for: Biochemical, Mineral, and Morphological Properties of Indian Tamarind (Tamarindus indica L.)
Source: Food Sci Nutr. 2026 Apr 12;14(4):e71754. doi: 10.1002/fsn3.71754 (PMC13071173; doi:10.1002/fsn3.71754)
Supplement: Supplementary file 1 — Table S1: Trait loadings for the first five principal components. [file FSN3-14-e71754-s001.docx]

**Supplementary Table S1.** Trait loadings for the first five principal components.

| **Trait** | **PC1** | **PC2** | **PC3** | **PC4** | **PC5** |
| --- | --- | --- | --- | --- | --- |
| Tree height (TH) | **−0.266** | −0.157 | −0.092 | 0.007 | 0.112 |
| Stem girth (SG) | −0.182 | −0.133 | −0.242 | 0.029 | −0.003 |
| Tree spread- East-West (TEW) | −0.247 | −0.169 | −0.144 | 0.028 | 0.069 |
| Tree spread- North-South (TNS) | −0.229 | −0.142 | −0.181 | 0.070 | 0.207 |
| Number of primary branches/tree(NPB) | 0.019 | 0.111 | −0.246 | −0.241 | −0.076 |
| Number of flower/inflorescence (NFI) | −0.111 | 0.211 | −0.049 | −0.032 | 0.088 |
| Pod length (PdL) | **−0.257** | −0.101 | 0.020 | 0.100 | 0.229 |
| Pod breadth (PdB) | −0.068 | −0.035 | **0.260** | 0.081 | 0.220 |
| Seed weight (SdWt) | −0.207 | 0.097 | −0.009 | **−0.253** | 0.120 |
| Number of seeds/pod (NSP) | −0.077 | 0.073 | −0.123 | **−0.285** | −0.044 |
| Shell weight (ShWt) | −0.223 | 0.162 | 0.062 | −0.157 | 0.229 |
| Fiber weight (FWt) | −0.034 | −0.162 | −0.151 | **−0.271** | 0.147 |
| Pulp weight (PWt) | −0.185 | **0.281** | 0.091 | −0.044 | 0.062 |
| Pulp: seed (PSR) | 0.051 | **−0.282** | −0.160 | −0.172 | −0.037 |
| Pulp percentage (PP) | −0.051 | **0.302** | 0.151 | 0.169 | −0.109 |
| Real pulp value (RPV) | −0.159 | **0.305** | 0.097 | 0.014 | 0.021 |
| Pod weight (PdWt) | −0.216 | 0.214 | 0.055 | −0.147 | 0.133 |
| Pod yield/tree (PdYT) | **−0.278** | −0.021 | −0.156 | 0.125 | −0.072 |
| Pod yield/ha (PdYH) | **−0.278** | −0.021 | −0.156 | 0.124 | −0.072 |
| Total soluble solids (TSS) | −0.136 | −0.016 | 0.056 | **0.246** | **−0.428** |
| Tartaric acid (TA) | −0.196 | −0.009 | −0.149 | −0.213 | −0.222 |
| Total sugars (TS) | −0.204 | −0.074 | −0.055 | 0.170 | **−0.364** |
| Reducing sugars (RS) | −0.092 | **−0.288** | 0.008 | 0.216 | 0.032 |
| Non-reducing sugars (NRS) | −0.079 | **0.253** | −0.057 | −0.088 | **−0.357** |
| Sweetness (TSA) | 0.150 | 0.040 | 0.084 | **0.291** | 0.092 |
| Vitamin C (VC) | −0.053 | −0.153 | **0.203** | 0.211 | 0.150 |
| Total phenols (TP) | −0.150 | −0.146 | **0.285** | −0.069 | −0.137 |
| Total flavonoids (TF) | −0.174 | −0.093 | **0.282** | −0.053 | −0.201 |
| Antioxidant activity (AA) | −0.191 | −0.144 | **0.252** | −0.026 | −0.156 |
| Potassium (K) | 0.030 | 0.222 | −0.068 | **0.249** | 0.184 |
| Phosphorus (P) | −0.032 | **0.277** | −0.219 | 0.143 | −0.072 |
| Calcium (Ca) | −0.045 | 0.133 | −0.235 | **0.268** | 0.021 |
| Magnesium (Mg) | −0.086 | 0.013 | **−0.293** | **0.273** | 0.084 |
| Sodium (Na) | 0.217 | 0.014 | −0.235 | 0.067 | 0.021 |
| Iron (Fe) | −0.218 | 0.113 | 0.190 | 0.032 | 0.186 |
